# β- and α_2_-Adrenoceptor Control of Vascular Tension and Catecholamine Release in Female Normotensive and Spontaneously Hypertensive Rats

**DOI:** 10.3389/fneur.2017.00130

**Published:** 2017-04-03

**Authors:** Torill Berg

**Affiliations:** ^1^Division of Physiology, Department of Molecular Medicine, Institute of Basic Medical Sciences, University of Oslo, Oslo, Norway

**Keywords:** hypertension, β-adrenoceptors, α_2_-adrenoceptors, norepinephrine, epinephrine, female rats, spontaneously hypertensive rats, total peripheral vascular resistance

## Abstract

As in humans, young, female, spontaneously hypertensive rats (SHR) have a lower blood pressure than male SHR. In male, normotensive rats (WKY), α_2_- and β_1+2_-adrenoceptors (AR) reciprocally controlled catecholamine release and vascular smooth muscle tension. This interaction was malfunctioning in male SHR. The present study analyzed if a favorable shift in the α_2_/β_1+2_AR interaction may represent an antihypertensive protection in females. Female SHR (early hypertension, 12–14 weeks) and age-matched WKY were infused with tyramine (15 min) to stimulate norepinephrine (NE) release through the reuptake transporter, consequently preventing reuptake. Presynaptic control of vesicular release was therefore reflected as differences in overflow to plasma. The released NE increased total peripheral vascular resistance (TPR). The results showed that β_1>2_AR facilitated tyramine-stimulated NE release in both strains, also in the presence of α_2_AR-antagonist (L-659,066). βAR-antagonist (atenolol-β_1_, ICI-118551-β_2_, nadolol-β_1+2_) had no effect on the increased secretion of epinephrine after L-659,066 in WKY, but β_1>2_AR-antagonist augmented the L-659,066-induced increase in the secretion of epinephrine in SHR. Nadolol increased the TPR response to tyramine with a greater effect in WKY than SHR, whereas β_1or2_-selective antagonists did not. One βAR-subtype may therefore substitute for the other. When both β_1+2_AR were blocked, α_2_AR-antagonist still reduced the TPR response in WKY but not SHR. Thus, α_2_/β_1+2_AR reciprocally controlled catecholamine release, with a particular negative β_1_AR-influence on α_2_AR-auto-inhibition of epinephrine secretion in SHR. Moreover, in these female rats, β_1/2_AR-independent α_2_AR-mediated vasoconstriction was seen in WKY but not SHR, but β_1/2_AR-mediated vasodilation downregulated adrenergic vasoconstriction, not only in WKY but also in SHR.

## Introduction

Blood pressure (BP) in premenopausal women and young spontaneously hypertensive rats (SHR) is lower than that in males of the same age (1, 2). In a previous study (3), systolic/diastolic blood pressure (SBP/DBP) in 12–14 weeks old, anesthetized SHR was measured to 183/146 and 108/75 mm Hg in males and females, respectively, the latter not significantly different from the 87/61 mm Hg recorded in female normotensive rats [Wistar Kyoto (WKY)] (3). The female SHR at this age may therefore be classified as prehypertensive, since female SHR of the same stock developed a high BP later in life, i.e., 170/129 mm Hg, which was not different from the 175/140 mm Hg in male SHR, both around 1-year old (3). The mechanism responsible for this gender-dependent difference in disease development is not known but may involve differences in the control of sympathetic nervous system activity and/or sympathetic control of vascular tension.

### Catecholamine Release

**Figure 1:**
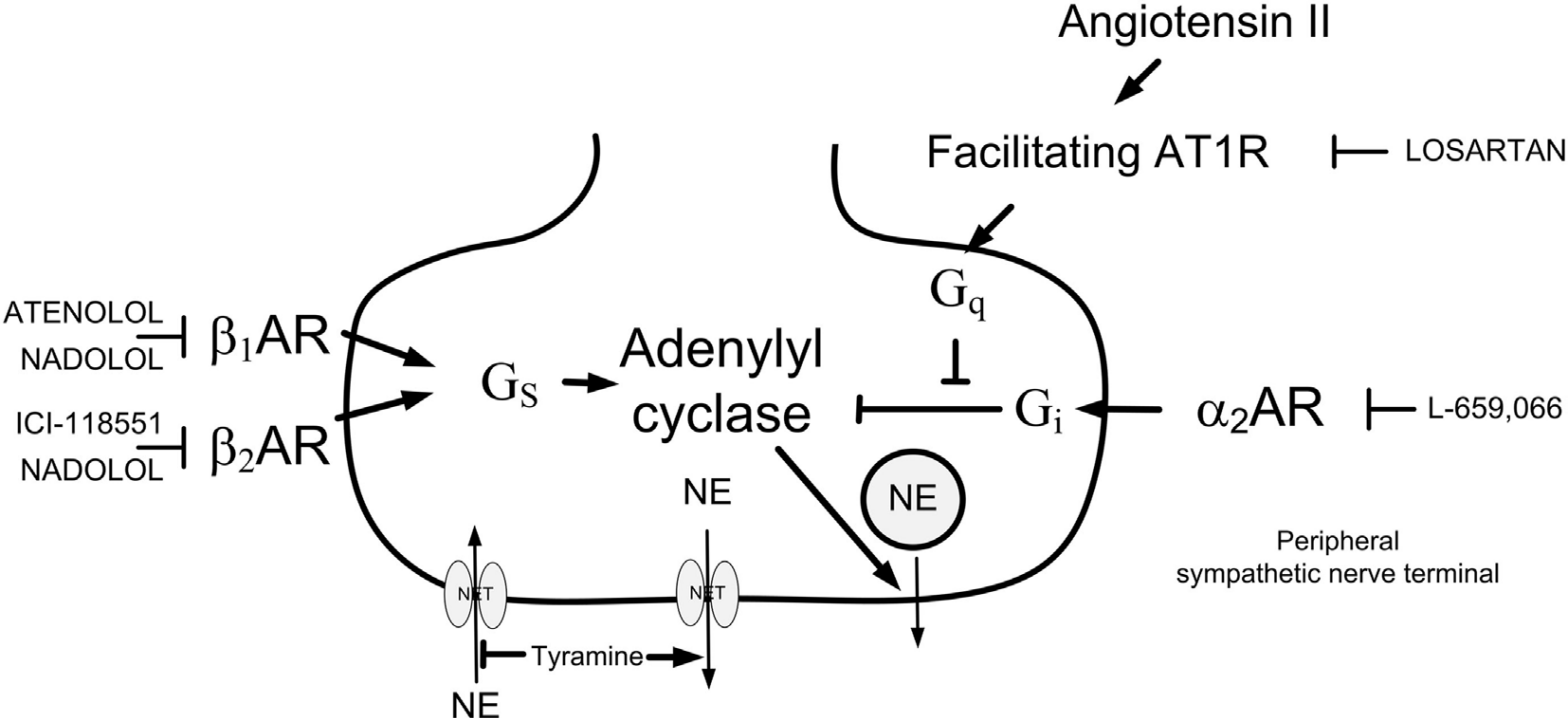
**Presynaptic modulation of vesicular norepinephrine (NE) release studied by the use of tyramine**. By reversing the transport through the norepinephrine reuptake transporter (NET), tyramine stimulates the release of NE, and through that prevents normal reuptake through NET. The released NE or other agonists present will activate presynaptic receptors, which will facilitate [β_1/2_AR and angiotensin AT1 receptor (AT1R)] or inhibit (α_2_AR) release of NE from synaptic vesicles. This influence will be reflected as differences in the NE overflow to plasma. Arrow—stimulatory action; blunted arrow—inhibitory action; G_s_—stimulatory G-protein; G_i_—inhibitory G-protein; G_q_—G_q_-protein.

The release of norepinephrine (NE) from sympathetic nerve endings is controlled by a reciprocal action of presynaptic α_2_AR and βAR, which inhibit and facilitate release, respectively (Figure 1). The α_2_AR-mediated auto-inhibition of release of NE and also epinephrine was functional in male WKY but not in the male SHR (4, 5). However, α_2_AR clearly inhibited release of both catecholamines in the female SHR (3). In male rats, both β_1_- and β_2_AR-facilitated release (5), and strain-related differences in the interaction between α_2_AR and β_1/2_AR were observed (6). The role of β_1+2_AR in the control of catecholamine release and their interaction with the α_2_AR has not been studied in the female SHR.

### Vascular Tension

Release of NE from peripheral sympathetic nerves evokes vasoconstriction by activating α_1_-adrenoceptors (AR) in the vascular smooth muscle cells (VSMC). This response is modulated reciprocally by βAR and α_2_AR, activating stimulatory (G_s_) and inhibitory (G_i_) G-proteins, respectively, opposing and enhancing the α_1_AR-mediated vasoconstriction. Thus, α_2_AR-antagonist lowered the rise in total peripheral vascular resistance (TPR) in response to endogenous release of NE in 12- to 14-week-old male WKY (4), and this reduction was in part counteracted by β_1/2/1+2_AR-antagonists (6). Dysfunctional α_2_AR has been observed in the male SHR (7, 8), and α_2_AR-antagonist had little effect on the rise in TPR during endogenous NE release in 12- to 14-week-old male SHR (4). Moreover, in male SHR, β_1+2_AR-mediated vasodilatation did not oppose NE-induced vasoconstriction even in the presence of α_2_AR-antagonist (6). However, in female SHR, α_2_AR-antagonist lowered the TPR response, although not equally efficient as in female WKY (3, 4). It is not known if the vasodilatory effect of α_2_AR-antagonist in female SHR in this early stage of hypertension is due to a more functional βAR-mediated vasodilatation than in male SHR. A gender-dependent difference in the β-AR control of vascular tension has been described in humans. In young men, muscle sympathetic nerve activity (MSNA) was correlated to TPR but inversely correlated to cardiac output (CO). Similar correlations were not observed in young women (9). This difference was attributed to βAR-mediated vasodilatation, since a positive correlation between MSNA and TPR was observed also in the women in the presence of the non-selective βAR-antagonist propranolol (10). An improved contribution from vasodilatory βAR may lower TPR. Since a high TPR is a hallmark of hypertension, a βAR-mediated downregulation of TPR may play a role in preserving a lower BP in the female gender.

The angiotensin AT1 receptor (AT1R) antagonist losartan lowered the TPR response to stimulated endogenous release of NE in male WKY but not SHR (11), suggesting that VSMC AT1R-signaling may depend on functional α_2_AR-G_i_ and/or βAR-G_s_ activation. Moreover, in male rats, losartan greatly increased the TPR and MBP responses to NE release when combined with atenolol, particularly in WKY (5). It is not known if this occurs also in female rats. Since losartan and atenolol are often used in combination to treat hypertension, this interaction may be of great importance for the outcome in situations with enhanced NE release such as during myocardial ischemia (12). A possible gender-dependent difference in this interaction may therefore be of clinical importance.

The purpose of the present study was therefore to determine the impact of β_1and2_AR on catecholamine release and vascular tension in female WKY and SHR. The second goal was to analyze the interaction between β_1and2_AR and α_2_AR and between the β_1_AR and AT1R in female rats. Differences between the present results on female rats compared to that previously observed in male rats will be discussed.

## Materials and Methods

### Experimental Procedure

The rats, i.e., WKY and SHR (Okamoto, SHR/NHsd strain) were originally supplied by formally legacy Harlan, now Envigo RMS, Bicester, England, and bred in-house. The rats included in this study were all 12- to 14-week-old females (67 WKY, 181 ± 2 g b.w., 13.0 ± 0.1 weeks; 67 SHR, 178 ± 1 g b.w., 12.9 ± 0.1 weeks). The rats were housed on 12/12 h light/dark cycles and were allowed food (conventional rat chow diet with 0.7% NaCl) and water *ad lib* until the time of the experiment. The rats were anesthetized with pentobarbital (70–75 mg/kg, i.p.) and tracheotomised. SBP and DBP were monitored through a catheter in the femoral artery, also used to record heart rate (HR) before the rats were connected to a positive-pressure ventilator. After thoracotomy, entering through the third intercostal space, CO (CO = minus cardiac flow) and HR were recorded by a flow probe on the ascending aorta, connected to a T206 Transonic Flow meter (Transonic Systems Inc., Ithaca, NY, USA). Mean arterial BP (MBP = SBP–DBP/3 + DBP) and TPR (MBP/CO) were calculated. The rats were ventilated with air throughout the experiment. Body temperature was maintained at 37–38°C by external heating, guided by a thermo sensor inserted inguinally about 1–2 cm into the abdominal cavity. All drugs were dissolved in phosphate-buffered saline (PBS = 0.01 M Na-phosphate, pH 7.4, 0.14 M NaCl) and administered through a catheter in the femoral vein. When all surgery was completed, the arterial catheter was flushed with 0.1 ml PBS containing 500 IU heparin/ml. The rats were injected with PBS as needed to stabilize BP and then allowed a resting period of 10 min.

### Experimental Design

All rats were infused with tyramine (1.26 μmol/min/kg, 15 min). Tyramine stimulates NE release by reversing the transport through the NE reuptake transporter (NET), consequently blocking reuptake (Figure 1). Activation of the presynaptic receptors by the released NE and/or other agonists/antagonists present will alter the vesicular release of NE, and this modification will be reflected as differences in the overflow of NE to plasma, as previously documented in detail (4, 13). The action of tyramine is peripherally restricted, i.e., tyramine does not cross the blood–brain barrier (14). Tyramine does not stimulate the secretion of epinephrine from the adrenal glands. However, the trauma induced by the surgical procedure activated some secretion of epinephrine, also subjected to receptor-mediated release control (3, 4, 13). Removal of the adrenal glands did not alter the plasma NE concentration, indicating that tyramine stimulated the release of NE from sympathetic nerves rather than from NE-producing cells in the adrenal medulla (5). The tyramine-induced NE release activated a cardiovascular response. The amount of catecholamines released into the synapse and into the circulation was most likely higher than that needed for a full cardiovascular response, and thus independent of drug-induced differences in catecholamine release. Antagonist-induced changes in the TPR response therefore reflected largely changes due to inhibition of the postsynaptic receptors.

**Figure 2:**
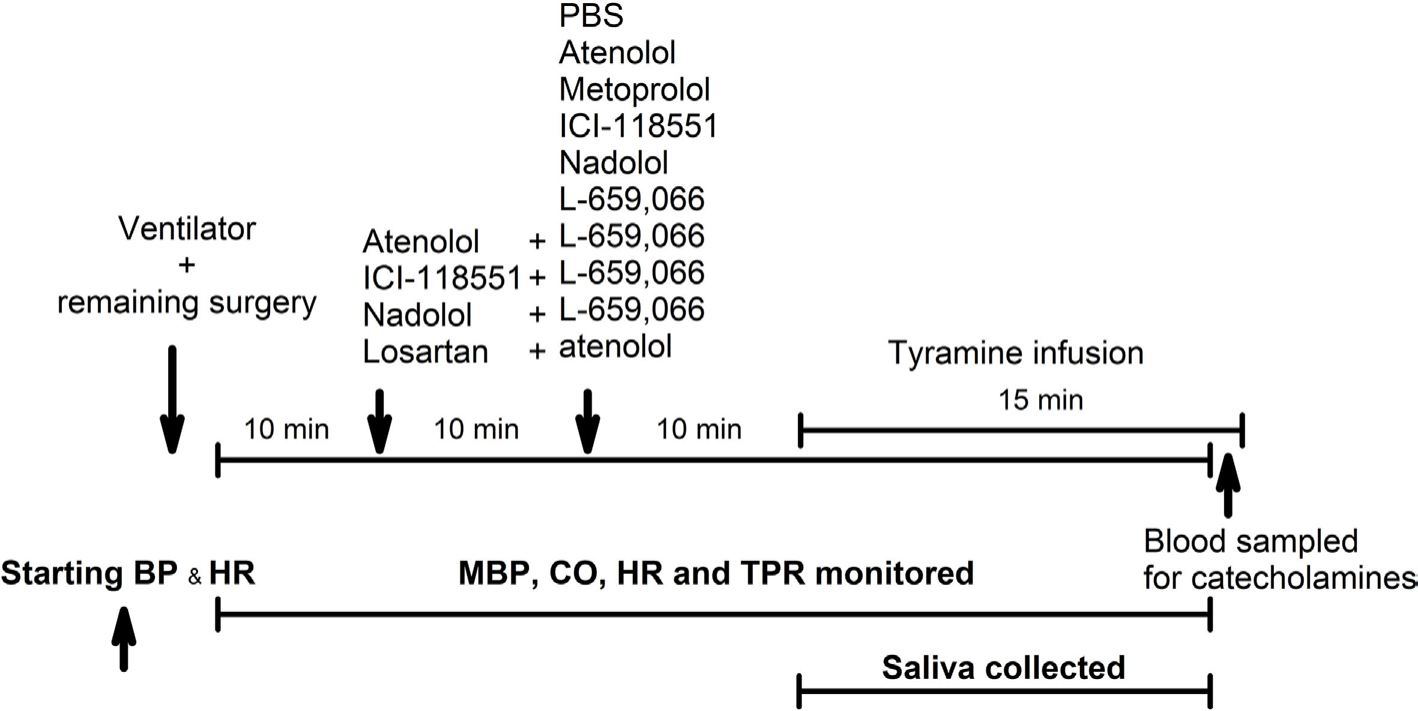
**An overview of the experimental design**. PBS—sham injection with vehicle. L-659,066—peripherally restricted and non-selective α_2_AR-antagonist. Atenolol and metoprolol—peripherally restricted and not restricted β_1_AR-antagonist, respectively. ICI-118551—not restricted β_2_AR-antagonist. Nadolol—peripherally restricted β_1+2_AR-antagonist. Losartan—angiotensin AT1 receptor antagonist. Tyramine—stimulates reverse transport of norepinephrine through the norepinephrine reuptake transporter.

A flowchart of the experimental design is shown in Figure 2. Control rats were pretreated with PBS, and the tyramine infusion was started 10 min later. To answer the questions if β_1_AR and/or β_2_AR stimulated catecholamine release and/or opposed the NE-induced rise in TPR, the PBS sham injection was substituted with either β_1_AR-selective antagonists, i.e., the peripherally restricted atenolol (5.6 μmol/kg) or the not restricted metoprolol (8.8 μmol/kg, β_1_), or the β_2_AR-selective antagonist ICI-118551 (initial dose of 1 μmol/kg, followed by 0.3 μmol/kg/min throughout the experiment) (15). To test for an additive effect of the β_1_AR and the β_2_AR, the rats were pretreated with the peripherally restricted β_1+2_AR-selective antagonist nadolol (8.5 μmol/kg) (15).

To test the influence of β_1_AR and β_2_AR on α_2_AR-mediated inhibition of catecholamine release and TPR, the rats were pretreated with atenolol, ICI-118551, or nadolol as above, followed 10 min later by the non-selective α_2_AR-antagonist L-659,066, which does not cross the blood–brain barrier (4, 13, 16).

To test for an interaction between β_1_AR and AT1R, rats were pretreated with the AT1R-antagonist losartan (79 μmol/kg) (17), followed 10 min later by atenolol and tyramine as above.

### Salivation

Salivation does not occur spontaneously in anesthetized rats but was stimulated here by tyramine. Saliva was collected with a pipette from the oral cavity throughout the tyramine-infusion period. Saliva volume was estimated by weight.

### Measurement of Plasma Catecholamines

Blood (1.5 ml) was collected from the arterial catheter after the tyramine-observation period, but without discontinuing the tyramine infusion (Figure 2). The blood was sampled into tubes containing 40 μl 0.2 M glutathione and 0.2 mol/l ethylene glycol-bis(2-aminoethylether)-*N,N,N*′,*N*′-tetraacetic acid (EGTA). Plasma was stored at −80°C until the catecholamine concentrations were determined using 400 μl plasma and the “5000 Reagent kit for HPLC analysis of Catecholamines in plasma” from Chromsystems GmbH, Munich, Germany, as previously described (6).

### Drugs

L-659,066 and losartan were kindly supplied by Merck, Sharp and Dohme Labs, Rahway, NJ, USA, ICI-118551 was obtained from ICI-Pharma, Cheshire, UK, and pentobarbital from The Norwegian National Hospital, Oslo, Norway. The remaining drugs were from Sigma Chemical Co., St. Louis, MO, USA.

### Statistical Analyses

Results are presented as mean values ± SEM. The cardiovascular data were averaged every min in all experiments, but every seven heartbeats (five samplings) for the starting BPs and HR. The cardiovascular response curves to tyramine were analyzed using Repeated Measures Analyses of Variance and Covariance, first as over-all tests including all groups or all groups within each strain, and subsequently for each group separately or between groups. When significant responses, differences, and/or interactions were indicated, significant responses were located at specific times using one-sample Student’s *t*-tests. Differences between groups at the same times were identified using two-sample Student’s *t*-tests for parametric data and Kruskal–Wallis tests for non-parametric data. For the MBP- and TPR response curves, these *ad hoc* analyses were done at the time of the TPR-peak response in the control groups, i.e., at 4 min, and also at 15 min. For the HR and CO responses, the *ad hoc* analyses were performed only at 15 min. The plasma catecholamine concentrations were first analyzed using two-way ANOVA, and the cardiovascular baselines, the effect of pretreatment and the tyramine-induced salivation by one-way ANOVA. Group- and strain-related differences were subsequently located by two-sample Student’s *t*-tests for parametric data, or, in the presence of outliers, by non-parametric Kruskal–Wallis tests. The *P*-value was for all tests and each step adjusted according to Bonferroni, except for differences in the effect of pretreatment, plasma catecholamine concentrations, and salivation, where *P* ≤ 0.05 was considered significant. Six to twelve rats were included in each group, based on sample power calculations using previous data from similar or related experiments. The rats in the WKY and SHR control and L-659,066-treated groups were in part the same as in a previous study (3), run intermittently with the present study.

## Results

### The Role of βAR and α_2_AR in the Control of Catecholamine Release in Female Rats

#### Norepinephrine

As before (3), the plasma concentration of NE at the end of the tyramine-infusion period was greater in SHR than in WKY (*P* = 0.001) (Table 1). The β_1_AR-antagonists atenolol and metoprolol reduced NE overflow to about 40–45% in both strains (*P* < 0.001), whereas the β_2_AR-antagonist ICI-118551 reduced the plasma concentration to 65–70% (*P* ≤ 0.013). The β_1+2_AR-antagonist nadolol reduced the plasma NE concentration to 58 and 45% in WKY and SHR, respectively (*P* < 0.001). The effect of ICI-118551 was less than that following β_1_AR-antagonists in both strains and in WKY also that of nadolol (*P* ≤ 0.015). As previously documented (3), the α_2_AR-antagonist L-659,066 increased the NE overflow in both strains (*P* = 0.026 and *P* < 0.001 in WKY and SHR, respectively). L-659,066 eliminated only in part the reduction induced by atenolol and ICI-118551 in WKY (*P* = 0.006 and 0.025 compared to βAR-antagonist alone), and the plasma concentration remained less than that in the controls (*P* ≤ 0.004). The inhibitory effect of atenolol on the L-659,066-induced increased NE release in WKY was greater than that of ICI-118551 (*P* = 0.003) but was not different from that of nadolol. In SHR, L-659,066 abolished the effect of βAR antagonist (*P* = NS compared to the controls) but remained less than that after L-659,066 alone when combined with atenolol or ICI-118551 (*P* ≤ 0.013). NE overflow in the nadolol + L-659,066-treated SHR was clearly higher than that after nadolol alone (*P* = 0.006) but not different from that after L-659,066 alone or that in the control group.

**Table 1:** **The plasma concentration of norepinephrine and epinephrine at the end of the tyramine-infusion period in female rats**.

	Normotensive rats (WKY)		Spontaneously hypertensive rats (SHR)	
	Norepinephrine (nM)	Epinephrine (nM)	Norepinephrine (nM)	Epinephrine (nM)
PBS + tyramine	24.0 ± 1.0	2.8 ± 1.1	32.8 ± 1.7*	2.2 ± 0.6
Metoprolol (β_1_AR) + tyramine	9.7 ± 1.2^†^	0.9 ± 0.3	16.5 ± 2.2^*,†^	6.9 ± 1.5^*,†^
Atenolol (β_1_AR) + tyramine	10.9 ± 0.6^†^	1.2 ± 0.6	15.9 ± 1.9^*,†^	5.2 ± 1.2^*,†^
ICI-118551 (β_2_AR) + tyramine	15.5 ± 1.5^†,├^	4.0 ± 1.1├	24.0 ± 2.5^*,†├^	10.4 ± 2.8^†^
Nadolol (β_1+2_AR) + tyramine	13.8 ± 0.6^†,├^	1.5 ± 0.7	14.7 ± 0.8^†^	5.9 ± 1.3^*,†^
L-659,066 (α_2_AR) + tyramine	34.4 ± 3.0^†^	15.3 ± 5.6^†^	45.6 ± 3.7^*,†^	15.4 ± 2.6^†^
Atenolol + L-659,066 + tyramine	14.7 ± 0.8^†,‡,§^	8.9 ± 1.5^†^	33.8 ± 2.6^*,‡,§^	41.8 ± 7.6^*,†,‡,§^
ICI-118551 + L-659,066 + tyramine	20.0 ± 1.0^†,‡,§,├^	13.4 ± 2.2^†^	30.8 ± 3.6^*,‡^	23.4 ± 4.1^†,§,├^
Nadolol + L-659,066 + tyramine	13.1 ± 0.7^†,‡^	13.8 ± 3.8^†,§^	37.4 ± 3.7^*,§^	41.3 ± 11.9^†,‡,§^
Losartan + atenolol + tyramine	10.9 ± 1.0^†^	1.7 ± 1.1	20.7 ± 1.5^*,†^	9.1 ± 2.1^†,*^

Differences were detected as indicated between corresponding WKY and SHR groups (* after SHR values), and within each strain between the controls and the experimental groups (^†^), between corresponding groups pretreated with L-659,066 and βAR-antagonist + L-659,066 (^‡^), between the groups pretreated with βAR-antagonist alone and corresponding βAR-antagonist + L-659,066 (^§^), between corresponding groups given β_1_AR-antagonist and β_2 or 1+2_AR-antagonist (^├^), or between groups given β_1_AR-antagonist + L-659,066 and β_2 or 1+2_AR-antagonist + L-659,066 (^├^), and between corresponding groups given atenolol and losartan + atenolol (none detected). ^*,†,‡,§,├^*P* ≤ 0.05.

Atenolol reduced the plasma NE concentration also when combined with losartan in both strains of female rats (*P* = NS compared to atenolol alone).

#### Epinephrine

A strain-related difference was not detected in the plasma epinephrine concentration in the control groups at the end of the experimental period (Table 1). β_1_-, β_2_-, and β_1+2_AR-antagonist alone had little effect on the plasma epinephrine concentration in WKY but slightly increased the concentration in SHR (*P* ≤ 0.044). L-659,066 increased the secretion of epinephrine in both strains (*P* ≤ 0.002). Atenolol, ICI-118551, and nadolol did not alter the enhancing effect of L-659,066 in WKY. However, atenolol and nadolol potentiated the effect of L-659,066 in SHR (*P* ≤ 0.039), whereas the increase observed after ICI-118551 was not statistically significant. Losartan + atenolol slightly increased the secretion of epinephrine in SHR.

### Cardiovascular Baselines in Female WKY and SHR and the Effect of Pretreatment

Starting BP and HR, recorded before the rats were connected to the respirator, were greater in the female SHR than in the female WKY (SBP/DBP/MBP = 138 ± 5/103 ± 4/115 ± 4 and 85 ± 3/60 ± 3/69 ± 3 mm Hg and HR = 373 ± 7 and 300 ± 9 bpm in SHR and WKY, respectively, *P* < 0.001, all groups included). As previously discussed (3, 4), SHR of both genders were more sensitive to the reduced venous return to the right heart during positive-pressure ventilation than WKY. Thus, after the rats were connected to the ventilator, surgery completed and the rats had been pretreated with PBS, i.e., prior to tyramine, strain-related differences in MBP and HR were no longer observed (Table 2). At this time, CO was lower, and TPR higher in SHR compared to WKY (*P* < 0.001).

**Table 2:** **Cardiovascular baselines after pretreatment, i.e., prior to tyramine, in female rats. The response to pretreatment is shown below in parenthesis**.

Pretreatment	Normotensive rats (WKY)				Spontaneously hypertensive rats (SHR)			
	MBP (mm Hg)	HR (bpm)	CO (ml/min)	TPR (mm Hg/ml/min)	MBP (mm Hg)	HR (bpm)	CO (ml/min)	TPR (mm Hg/ml/min)
PBS	64 ± 3 (−3 ± 2)	356 ± 12 (0 ± 6)	22 ± 1 (1 ± 0)	2.9 ± 0.1 (−0.2 ± 0.1)	58 ± 3 (−16 ± 5)*	365 ± 8 (−32 ± 5)*	14 ± 1* (−1 ± 0)*	4.3 ± 0.4* (−0.7 ± 0.3)
Atenolol	54 ± 3^†^ (−5 ± 3)	324 ± 6^†^ (−23 ± 8)^†^	20 ± 1 (−1 ± 1)	2.8 ± 0.1 (−0.1 ± 0.0)	63 ± 3 (−18 ± 7)	350 ± 14 (−60 ± 12)^†^	12 ± 1 (−2 ± 1)	5.4 ± 0.2^†^ (−0.4 ± 0.3)
Metoprolol	67 ± 3 (−6 ± 3)	331 ± 6 (−32 ± 7)^††^	22 ± 2 (−1 ± 1)	3.2 ± 0.3 (−0.2 ± 0.2)	55 ± 3 (−17 ± 3)	301 ± 5^††^ (−84 ± 11)^††^	13 ± 1 (−1 ± 1)	4.2 ± 0.2 (−1.3 ± 0.6)
ICI-118551	55 ± 4 (−1 ± 3)	334 ± 12 (−20 ± 8)	22 ± 1 (−2 ± 1)	2.5 ± 0.2 (0.1 ± 0.1)^†^	50 ± 4 (−20 ± 6)	318 ± 10^††^ (−65 ± 11)^†^	15 ± 1 (−3 ± 1)	3.4 ± 0.1^†^ (−0.6 ± 0.3)
Nadolol	55 ± 4 (−3 ± 4)	337 ± 6 (−21 ± 7)^†^	20 ± 2 (−2 ± 1)^†^	3.0 ± 0.3 (0.3 ± 0.2)^†^	53 ± 4 (−39 ± 10)	315 ± 13^†^ (−92 ± 15)^††^	13 ± 0 (−3 ± 1)^†^	4.2 ± 0.4 (−1.5 ± 0.4)
L-659,066	54 ± 3 (−9 ± 2)	360 ± 7 (−5 ± 4)	24 ± 2 (−1 ± 0)	2.3 ± 0.3^†^ (−0.3 ± 0.1)	57 ± 6 (−25 ± 4)	347 ± 8 (−34 ± 6)	12 ± 1 (−4 ± 1)	5.3 ± 1.5 (0.2 ± 0.9)
Atenolol + L-659,066	48 ± 2^†^ (−12 ± 4)	339 ± 5 (−29 ± 9)^†^	23 ± 1 (0 ± 1)	1.9 ± 0.1^†^ (−0.7 ± 0.1)^†^	54 ± 4 (−28 ± 5)	303 ± 8^††^ (−88 ± 6)^††^	13 ± 1 (−1 ± 1)	3.4 ± 0.3 (−0.9 ± 0.2)
ICI-118551 + L-659,066	48 ± 5^†^ (−5 ± 2)	333 ± 11 (−24 ± 9)^†^	21 ± 2 (0 ± 1)	2.3 ± 0.1^†^ (−0.6 ± 0.1)	42 ± 3^††^ (−20 ± 3)	283 ± 13^††^ (−85 ± 5)^††^	15 ± 1 (−2 ± 1)	4.4 ± 0.4 (−1.7 ± 0.4)
Nadolol + L-659,066	58 ± 3 (1 ± 1)	334 ± 7 (−25 ± 5)^††^	24 ± 1 (2 ± 1)	2.5 ± 0.2 (−0.1 ± 0.1)	42 ± 2^††^ (−26 ± 10)	317 ± 10^††^ (−99 ± 17)^††^	15 ± 2 (−3 ± 1)	3.3 ± 0.2^†^ (−1.0 ± 0.4)
Losartan + atenolol	54 ± 1^††^ (−18 ± 3)^††^	345 ± 10 (−15 ± 6)	27 ± 5 (2 ± 1)	2.2 ± 0.3^††^ (−0.9 ± 0.2)^††^	39 ± 1^††^ (−36 ± 5)^†^	291 ± 5^††^ (−106 ± 11)^††^	9 ± 1^††^ (−5 ± 1)^††^	4.5 ± 0.3 (−0.9 ± 0.4)

Comparisons were made between the WKY and SHR controls (* after SHR values) and between the PBS control and the experimental groups within each strain (^†^). *P ≤ 0.0125, ^†^*P* ≤ 0.05, ^††^*P* ≤ 0.0056.

The cardiovascular response to pretreatment is shown in Table 2. The major findings were that L-659,066 and atenolol + L-659,066 reduced TPR (*P* ≤ 0.024) and losartan + atenolol reduced both MBP and TPR (*P* ≤ 0.005) in WKY, whereas TPR was slightly higher after ICI-118551 or nadolol (*P* ≤ 0.033) in this strain. In SHR, MBP was reduced by ICI-118551 + L-659,066, nadolol + L-659,066, and losartan + atenolol, and TPR by atenolol + L-659,066 (*P* ≤ 0.021). Baseline HR was reduced in all groups given βAR antagonist, alone or combined with L-659,066 (*P* ≤ 0.044) in both strains, except in the WKY ICI-118551 and losartan + atenolol groups. Nadolol and losartan + atenolol reduced also CO in SHR (*P* < 0.001).

### The Effect of βAR-Antagonists on the Cardiovascular Response to Tyramine and Their Interaction with α_2_AR-Antagonist in Female Rats

**Figure 3:**
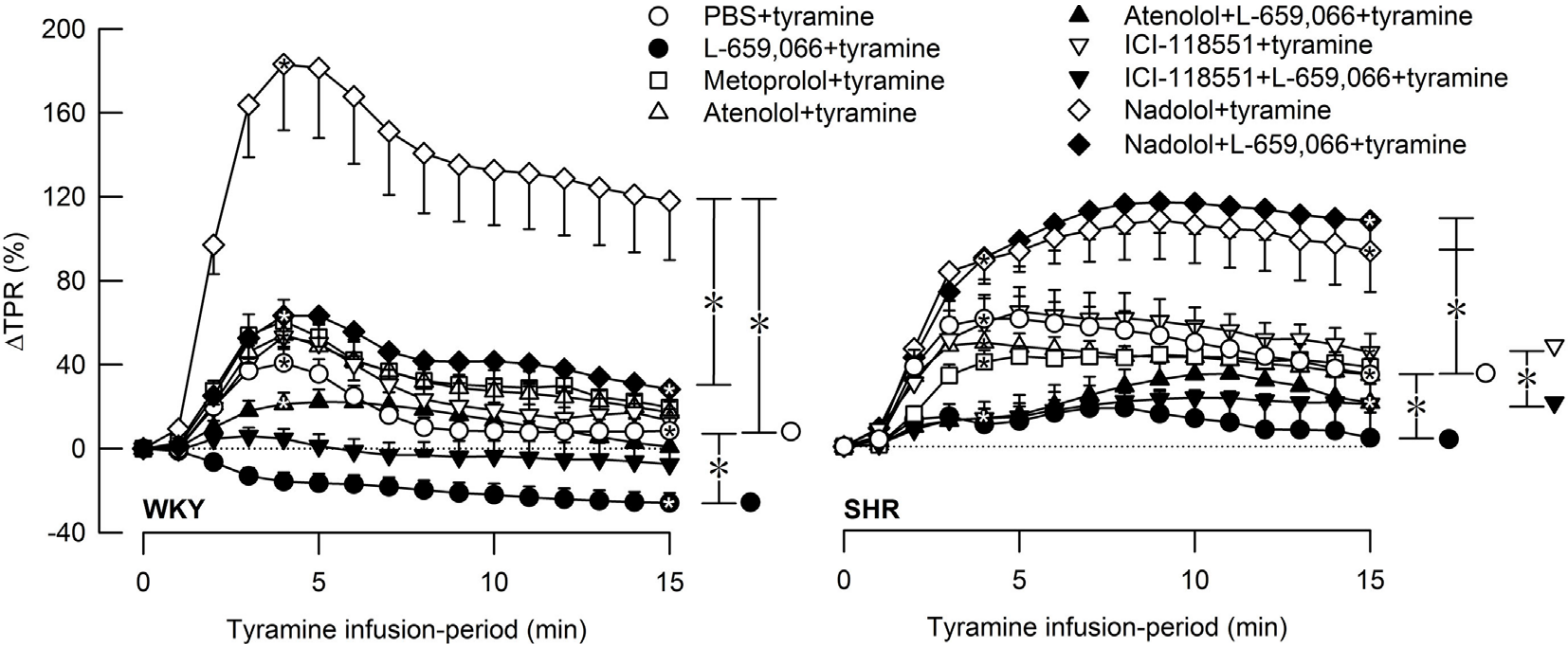
**The total peripheral vascular resistance (TPR) response to tyramine-induced norepinephrine release in female normotensive (WKY) and spontaneously hypertensive rats (SHR)**. The rats were pretreated with β_1_- (metoprolol and atenolol), β_2_- (ICI-118551), β_1+2_AR- (nadolol) or α_2_AR- (L-659,066) antagonists, alone or combined, as indicated by the symbol legends. Baselines prior to tyramine are shown in Table 2. Significant responses (one-sample Student’s *t*-tests, * within symbol) and differences between the control and experimental groups (two-sample Student’s *t*-tests, * in brackets) were located at 4 min (peak response, please see Figure 4) and at 15 min (brackets right of curves). Comparisons were also made between βAR-antagonist alone and the L-659,066 + βAR-antagonist groups. *P ≤ 0.025 for one- and two-sample Student’s *t*-tests after curve evaluations using Repeated Measures Analyses of Variance and Covariance (see Materials and Methods).

**Figure 4:**
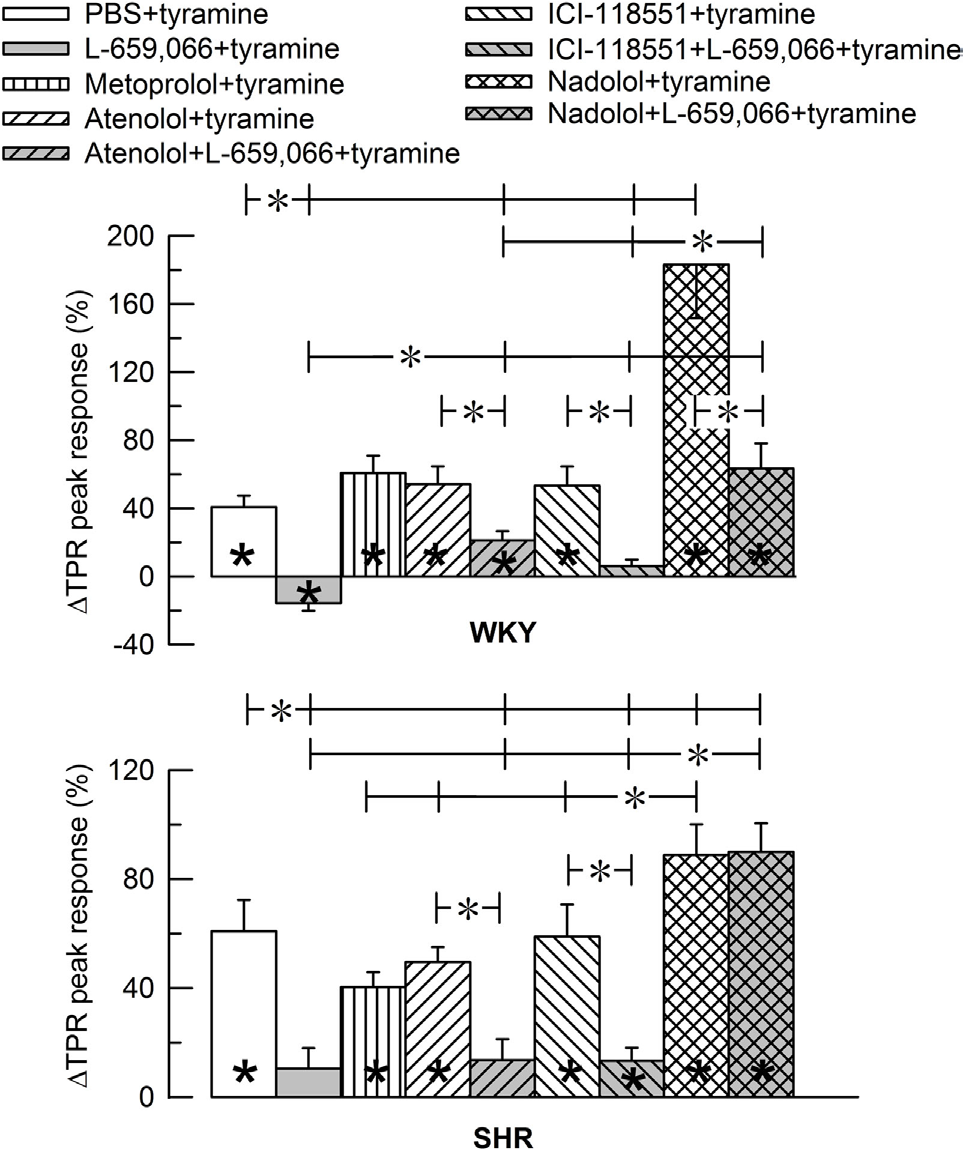
**Bar graph of the total peripheral vascular resistance (TPR)-peak response to tyramine in the experiments shown in Figure 3**. The female normotensive (WKY) and spontaneously hypertensive rats (SHR) were pretreated with β_1_- (metoprolol and atenolol), β_2_- (ICI-118551), β_1+2_AR- (nadolol) or α_2_AR- (L-659,066) antagonists, alone or combined, as indicated by the symbol legends. Significant responses (* within column) and differences between groups (two-sample Student’s *t*-tests, * in brackets) were located as indicated. Comparisons were made between the control and the experimental groups, between corresponding groups given L-659,066 or βAR-antagonist alone and the L-659,066 + βAR-antagonist groups and between the nadolol + L-659,066 and β_1/2_AR + L-659,066 groups. Please notice the difference in the scale in the two graphs. *P ≤ 0.025 tests after curve evaluations using Repeated Measures Analyses of Variance and Covariance (see Materials and Methods).

**Figure 5:**
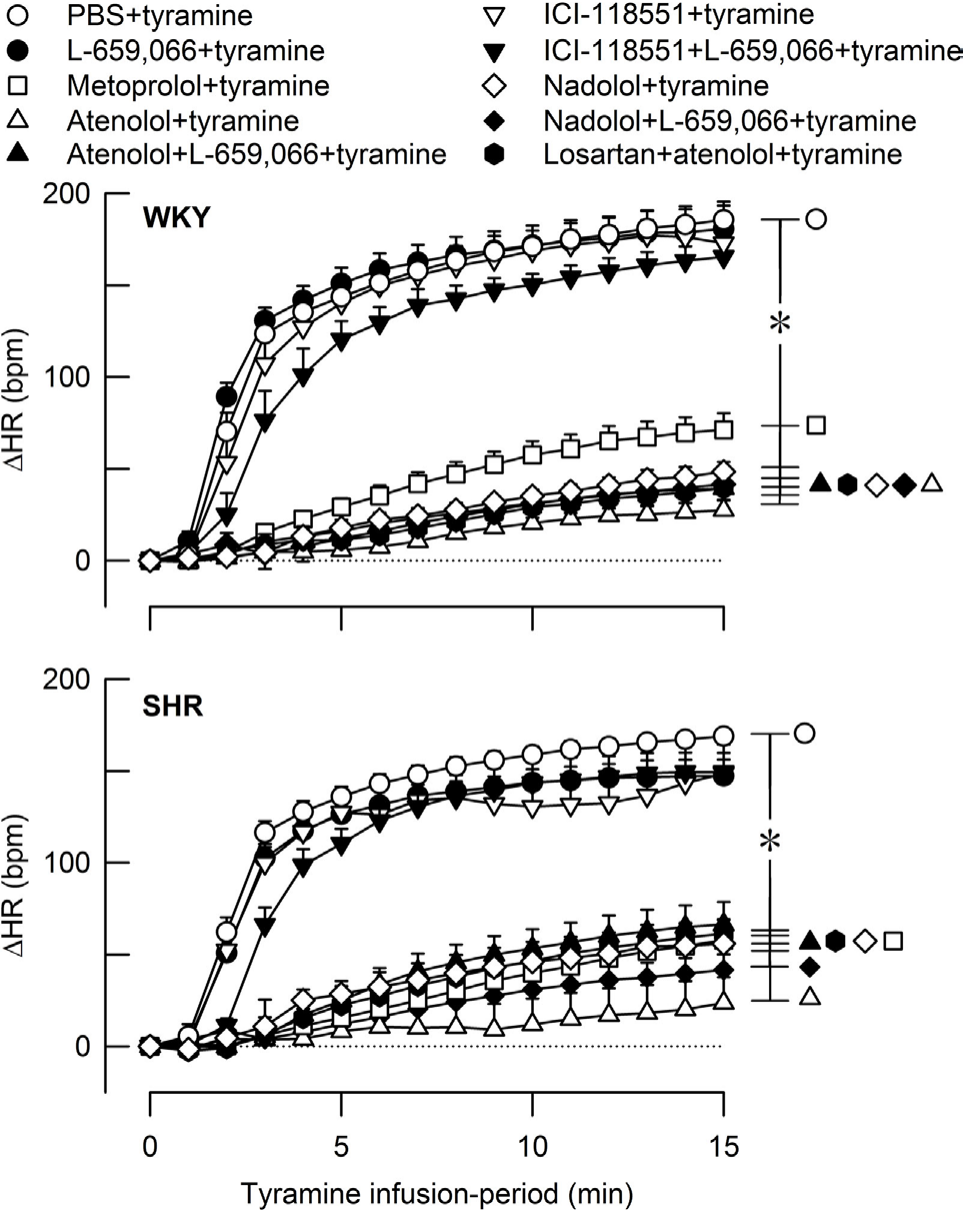
**The heart rate (HR) response to tyramine-induced norepinephrine release in female normotensive (WKY) and spontaneously hypertensive rats (SHR)**. The rats were pretreated with β_1_- (metoprolol and atenolol), β_2_- (ICI-118551), β_1+2_AR- (nadolol), α_2_AR- (L-659,066) or angiotensin AT1 receptor antagonists, alone or combined, as indicated by the symbol legends. Baselines prior to tyramine are shown in Table 2. The change in HR after 15 min was significant in all groups (one-sample Student’s *t*-tests, not indicated). Significant differences between the control and experimental groups (two-sample Student’s *t*-tests, * in brackets right of curves) were located at 15 min as indicated. *P ≤ 0.05 after curve evaluations using Repeated Measures Analyses of Variance and Covariance (see Materials and Methods).

**Figure 6:**
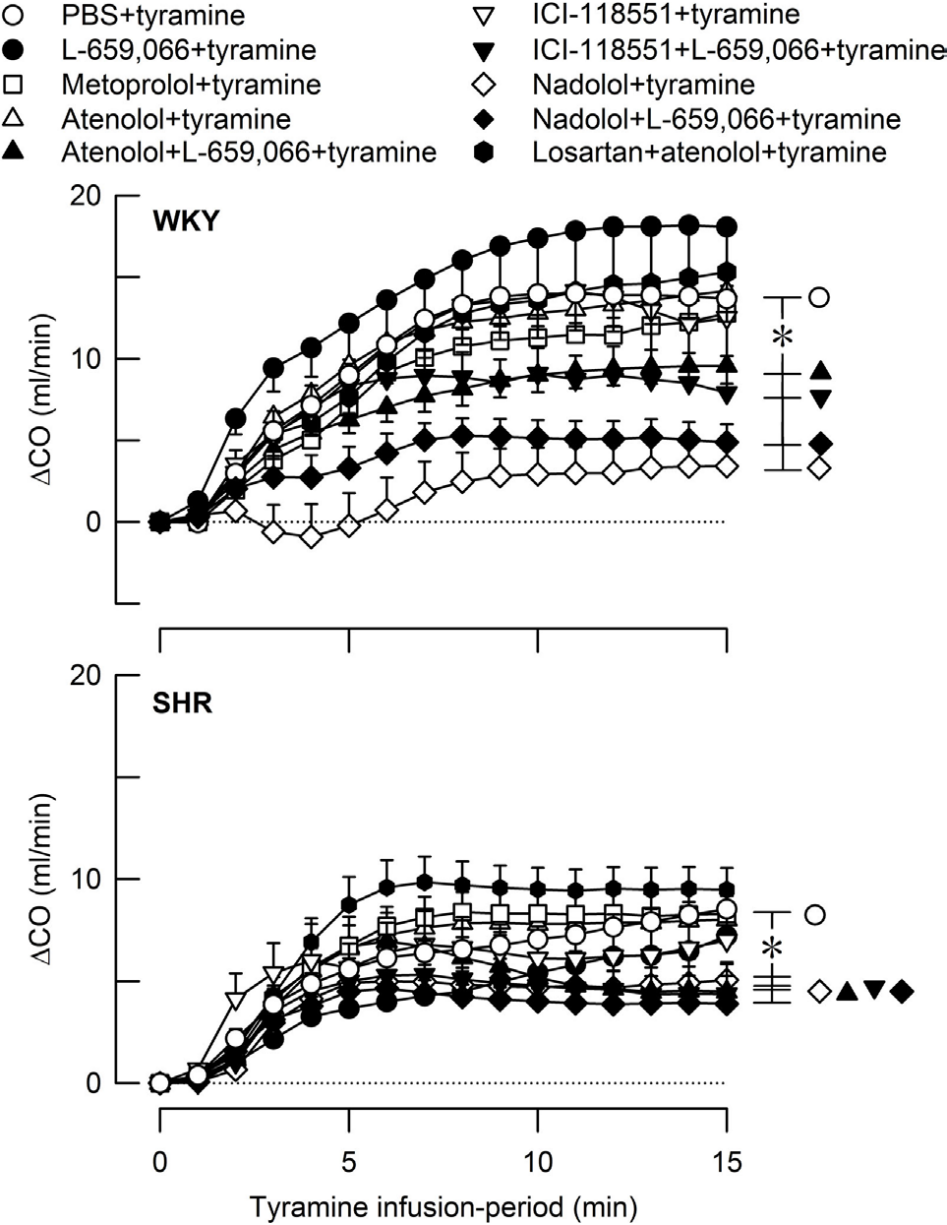
**The cardiac output (CO) response to tyramine-induced norepinephrine release in female normotensive (WKY) and spontaneously hypertensive rats (SHR)**. The rats were pretreated with β_1_- (metoprolol and atenolol), β_2_- (ICI-118551), β_1+2_AR- (nadolol), α_2_AR- (L-659,066) or angiotensin AT1 receptor antagonists, alone or combined, as indicated by the symbol legends. Baselines prior to tyramine are shown in Table 2. The change in CO after 15 min was significant in all groups (one-sample Student’s *t*-tests, not indicated). Significant differences between the control and the experimental groups were located at 15 min as indicated (two-sample Student’s *t*-tests, * in brackets right of curves). *P ≤ 0.05 after curve evaluations using Repeated Measures Analyses of Variance and Covariance (see Materials and Methods).

**Figure 7:**
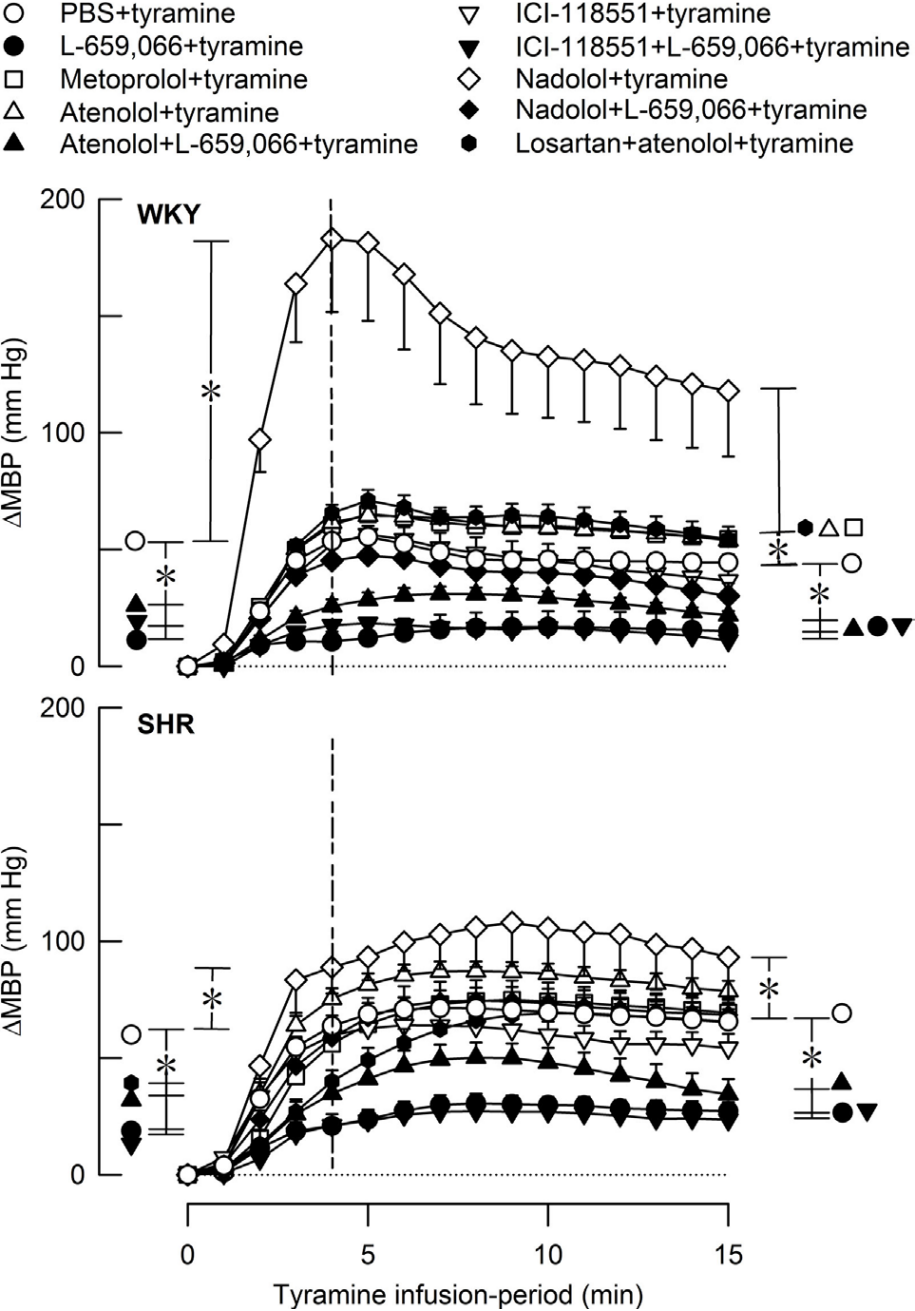
**The MBP response to tyramine-induced norepinephrine release in female normotensive (WKY) and spontaneously hypertensive rats (SHR)**. The rats were pretreated with β_1_- (metoprolol and atenolol), β_2_- (ICI-118551), β_1+2_AR (nadolol), α_2_AR- (L-659,066) or angiotensin AT1 receptor antagonists, alone or combined, as indicated by the symbol legends. Baselines prior to tyramine are shown in Table 2. The change in MBP at 4 min, i.e., at the total peripheral vascular resistance (TPR)-peak response (dotted vertical lines), and at 15 min was significant in all groups (one-sample Student’s *t*-tests, not indicated). Significant differences between the control and experimental groups were located at the TPR-peak response and 15 min as indicated (two-sample Student’s *t*-tests, * in brackets left and right of curves, respectively). *P ≤ 0.025 after curve evaluations using Repeated Measures Analyses of Variance and Covariance (see Materials and Methods).

As previously documented (3), the tyramine-stimulated release of NE induced a rise in TPR, which reached a peak after 4 min in both strains (Figures 3 and 4), and a sustained increase in HR (Figure 5), CO (Figure 6), and MBP (Figure 7). The TPR response to tyramine was transient in WKY, but sustained in SHR, and was higher in SHR than in WKY at the end of the tyramine-infusion period (*P* ≤ 0.015).

The effect of βAR- and α_2_AR-antagonists on the TPR response throughout the tyramine-infusion period is shown in Figure 3 and on the immediate TPR-peak response as bar graphs in Figure 4. Nadolol (*P* ≤ 0.007), but not metoprolol, atenolol, or ICI-118551 (*P* = NS), increased the immediate and late TPR response to tyramine in both WKY and SHR. The effect of nadolol during the immediate response to tyramine was greater in WKY than in SHR (*P* = 0.016). L-659,066 changed the vasoconstriction to a vasodilatory response in WKY and eliminated the vasoconstriction in SHR. The L-659,066-dependent vasodilatation in WKY was reversed to vasoconstriction after additional pretreatment with atenolol but remained lower than that in the WKY control group (*P* ≤ 0.008 compared to the L-659,066 or atenolol-only groups or the controls, at 4 min). After pretreatment with ICI-118551 + L-659,066, the vasodilatory response was eliminated but not changed to vasoconstriction (*P* ≤ 0.007 compared to the L-659,066- or ICI-118551-only groups or the WKY control group, at 4 min). After nadolol + L-659,066, the immediate and late TPR response to tyramine in WKY was lower than that after nadolol alone (*P* ≤ 0.011) and not different from that in the controls, but higher than that after atenolol/ICI-118551 + L-659,066 and after L-659,066 alone (*P* ≤ 0.016). In SHR, the TPR response to tyramine after pretreatment with atenolol + L-659,066 or ICI-118551 + L-659,066 was not different from that after L-659,066 alone. The elevated TPR response after nadolol was not influenced by additional pretreatment with L-659,066 in SHR, and ΔTPR in SHR pretreated with nadolol + L-659,066 was higher than that in the controls and after L-659,066 alone and after atenolol/ICI-118551 + L-659,066 (*P* ≤ 0.001).

**Figure 8:**
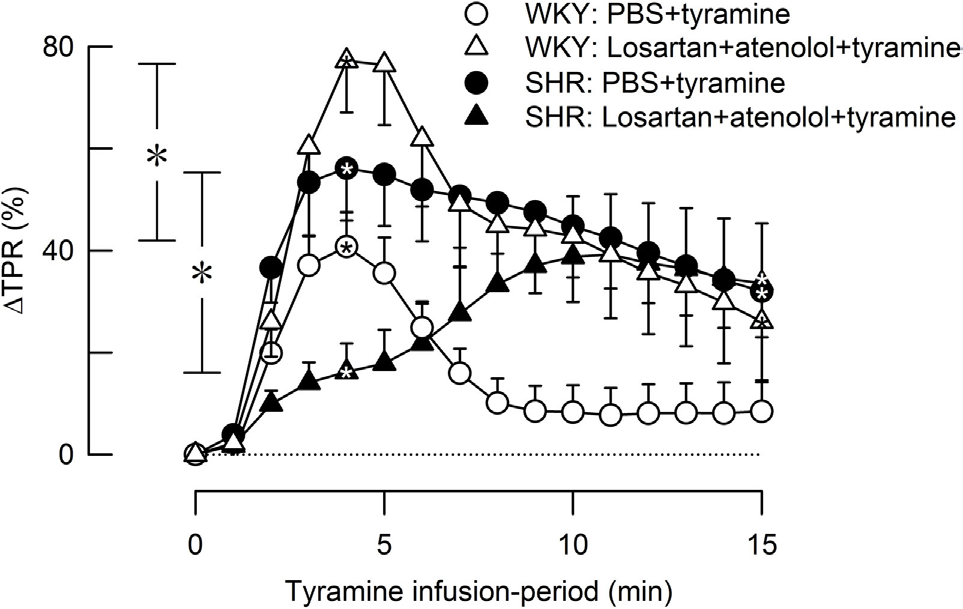
**The effect of losartan combined with atenolol on total peripheral vascular resistance (TPR) response to tyramine-induced norepinephrine release in female normotensive (WKY) and spontaneously hypertensive rats (SHR)**. Baselines prior to tyramine are shown in Table 2. Significant responses (one-sample Student’s *t*-tests, * within symbol) and group differences (two-sample Student’s *t*-tests) were located as indicated at 4 min (peak response, * in brackets left of curves) and at 15 min (* in brackets right of curves). Comparisons were made between corresponding control and losartan + atenolol-treated groups. *P ≤ 0.025 after curve evaluations using Repeated Measures Analyses of Variance and Covariance (see Materials and Methods).

Losartan + atenolol strongly increased the TPR-peak response to tyramine in female WKY (*P* = 0.008) but reduced the response in SHR (*P* = 0.004) (Figure 8).

A strain-related difference was not observed in the tyramine-induced tachycardia (*P* = NS) (Figure 5). The HR response to tyramine was clearly reduced in all groups where pretreatment contained a β_1_AR-antagonistic component but was not influenced by the β_2_-selective antagonist ICI-118551. L-659,066 alone had no effect on the tyramine-induced tachycardia, and L-659,066 did not alter the effect of βAR-antagonist on this response. The tachycardia after losartan + atenolol was not different from that after atenolol alone.

Tyramine also increased CO, and with a greater effect in female WKY than in female SHR (Figure 6). However, due to the lower baseline in SHR, the strain-related difference was not seen when the increase was expressed in percent of baseline (ΔCO after 15 min = 64 ± 7 and 64 ± 8% in WKY and SHR, respectively, *P* = NS). The tyramine-induced rise in CO was reduced in both strains after nadolol alone and after atenolol/ICI-118551/nadolol + L-659,066 (*P* ≤ 0.01 at 15 min).

The rise in MBP at the end of the tyramine-infusion period was slightly higher in SHR than in WKY (*P* = 0.003) (Figure 7). The MBP response in WKY was greatly increased after nadolol (*P* < 0.001), and atenolol and metoprolol slightly increased the late MBP response in this strain. The βAR antagonists had no significant effect on the MBP response in SHR. L-659,066, alone or combined with atenolol or ICI-118551, reduced the MBP response throughout the infusion period in both strains (*P* ≤ 0.006). The enhanced MBP response after nadolol in WKY was eliminated by additional pretreatment with L-659,066 (*P* < 0.001) and was no longer different from that in the WKY control group. Losartan + atenolol slightly enhanced the MBP response to tyramine in WKY, whereas losartan + atenolol reduced the initial response in SHR.

### The Role of βAR and α_2_AR in the Tyramine-Induced Salivation

Tyramine stimulated salivary secretion. Salivation was abolished in all groups pretreated with β_1_AR-antagonist or nadolol, reduced or eliminated after ICI-118551 + L-659,066 and nadolol + L-659,066, but was not influenced by β_2_-selective antagonist alone (Table 3).

**Table 3:** **Tyramine-induced salivation in female rats**.

Pretreatment	Normotensive rats (WKY) (μl)	Spontaneously hypertensive rats (SHR) (μl)
PBS	24 ± 7	16 ± 4
Atenolol	0 ± 0*	0 ± 0*
Metoprolol	0 ± 0*	0 ± 0*
ICI-118551	18 ± 9	6 ± 1
Nadolol	2 ± 2*	0 ± 0*
L-659,066	9 ± 2	11 ± 3
Atenolol + L-659,066	0 ± 0*	0 ± 0*
ICI-118551 + L-659,066	3 ± 3*	3 ± 2*
Nadolol + L-659,066	0 ± 0*	2 ± 2*
Losartan + atenolol	0 ± 0*	0 ± 0*

Comparisons were made between the PBS control and the experimental groups within each strain (*). The difference between the WKY and SHR control groups was not statistically significant. *P ≤ 0.05.

## Discussion

The main findings in the present study on female rats were that β_1>2_AR facilitated tyramine-stimulated NE release in WKY and SHR, whereas β_1>2_AR opposed α_2_AR auto-inhibition of epinephrine secretion in SHR but not WKY. Second, a strong β_1+2_AR-mediated vasodilatation counteracted NE-induced vasoconstriction in female WKY. β_1+2_AR-antagonist counteracted NE-induced vasoconstriction also in the female SHR, although less than that in WKY. α_2_AR-antagonist still lowered the TPR response to tyramine in the presence of β_1+2_AR-antagonist in WKY but not in SHR.

### Control of Catecholamine Release in Female Rats and a Comparison with That Previously Observed in Male Rats

#### Norepinephrine

Tyramine-stimulated overflow of NE in female rats was higher in SHR than in WKY (present study), similar to that previously observed in male rats (5, 6). As in male rats (5), β_1_AR efficiently facilitated NE release in both strains of female rats. This was concluded since atenolol and metoprolol both reduced the overflow of NE to plasma by 50–60%. Atenolol, unlike metoprolol, does not cross the blood–brain barrier, but the influence of the two antagonists on NE overflow did not differ. The effect therefore appeared to be peripheral, most likely involving presynaptic β_1_AR which facilitated the release of NE, as previously documented in detail in male rats (5).

ICI-118551 reduced the tyramine-induced NE overflow in both strains in female rats, demonstrating that also β_2_AR facilitated NE release. This was similar to that previously seen in male rats (5). The effect of the β_2_AR-antagonist was less than that after β_1_AR-antagonist in female WKY and SHR (present study), as in the male WKY (5), but not in male SHR where the two antagonists had the same effect (5). The greater effect of atenolol compared to ICI-118551 was likely to reflect that tyramine activated massive release of NE. NE was therefore present in a higher concentration in the area around the presynaptic receptors than epinephrine, and NE has the same affinity for the β_1_AR as epinephrine (18). Pretreatment with the β_1+2_AR-antagonist nadolol demonstrated that the two βAR did not have an additive effect in female WKY or SHR, similar to that previously observed in male rats (6). One βAR-subtype therefore appeared to substitute for the other in both genders and in both strains.

α_2_AR-mediated auto-inhibition of release was observed in female rats of both strains, demonstrated by the increased tyramine-induced overflow of NE to plasma after pretreatment with L-659,066. This differed from that previously observed in male rats, where L-659,066 increased the plasma NE concentration in WKY but not significantly in SHR (4). In the presence of L-659,066, β_1_-, β_2_-, and β_1+2_AR-antagonist still reduced NE overflow in both strains in the female rats, although apparently with a greater effect in WKY than in SHR. Also this observation differed from that seen in male rats, where ICI-188551 and nadolol reduced NE overflow in male WKY when combined with L-659,066, whereas atenolol did not (6), indicating that α_2_AR-signaling was a required substrate for β_1_AR-mediated stimulation of release in the male WKY. However, in male SHR, the presence of β_1_- but not β_2_AR-antagonist restored α_2_AR function, i.e., L-659,066 clearly increased NE overflow in the presence of atenolol or nadolol but not ICI-11855 (6). β_1_AR-mediated facilitation of release therefore opposed α_2_AR-mediated inhibition of NE release in the male but not in the female SHR. This observation may explain the dysfunctional α_2_AR-auto-inhibition of NE release in male SHR. The β_1_AR-mediated facilitation of release was not influenced by presynaptic release-stimulating AT1R (Figure 1) in female rats of either strain (present study), similar to that previously seen in male rats (5).

#### Epinephrine

The secretion of epinephrine from the adrenal glands was not activated by tyramine but by the trauma induced by the experiment itself (19). The control of this secretion was dominated by α_2_AR-auto-inhibition in both strains in the female rats. This conclusion was based on the fact that L-659,066 increased the secretion of epinephrine in both strains, whereas the βAR-antagonists alone had no effect in WKY and slightly increased the concentration in SHR. In the male rats, a significant α_2_AR-auto-inhibition of epinephrine secretion was regularly seen in WKY and occasionally in SHR (4, 6, 11). However, atenolol, ICI-118551, and nadolol potentiated the effect of the α_2_AR-antagonist in the female SHR (present study) similar to that seen in male WKY and SHR (6), in general with a greater effect of the β_1_- than the β_2_AR-antagonist and with a greater increase in the plasma epinephrine concentration in SHR than in WKY. However, none of the βAR-antagonists potentiated α_2_AR function in the female WKY. Atenolol combined with AT1R-antagonist had no effect on the secretion of epinephrine. It was therefore concluded that βAR, with a greater effect of the β_1_- than the β_2_-subtype, opposed α_2_AR auto-inhibition of epinephrine secretion in all rats except the female WKY. The importance of this observation was not clear, since the role of epinephrine in the pathogenesis of hypertension is not really known. However, a failing α_2_AR-mediated inhibition of adrenal epinephrine release has been shown to increase the concentration of circulating catecholamines with a detrimental effect on the outcome of myocardial infarction in mice (20). It may be assumed that an improved α_2_AR control of adrenal catecholamine release after β_1_AR-blocker may be beneficial from the viewpoint of lowering catecholamine release.

### βAR- and α_2_AR-Mediated Control of Vascular Tension in Female Rats

In female rats, βAR-mediated vasodilatation downregulated the vasoconstrictory TPR response to tyramine-stimulated NE release in both strains. This was indicated by the greatly enhanced TPR response throughout the tyramine-infusion period after pretreatment with the peripherally restricted β_1+2_AR-antagonist nadolol. The effect of nadolol was greater in WKY than in SHR, but still a clear effect of nadolol was observed in the female SHR.

The α_2_AR-antagonist L-659,066 reversed the vasoconstrictory TPR response to tyramine to a vasodilatory response in female WKY and eliminated the vasoconstriction in the female SHR. Nadolol fully reversed this reduction in TPR in female WKY. Inhibition of α_2_AR therefore unshielded a βAR-mediated vasodilation, which clearly opposed the NE-induced vasoconstriction in female WKY. However, the TPR response to tyramine in female WKY pretreated with nadolol + L-659,066 was far less than that after nadolol alone, suggesting that α_2_AR may mediate vasoconstriction also through a mechanism other than inhibition of the βAR-adenylyl cyclase stimulation. On the other hand, L-659,066 did not lower the enhanced TPR response to tyramine after nadolol in female SHR. It therefore seemed that α_2_AR-mediated vasoconstriction depended exclusively on βAR-mediated vasodilation as a substrate in the female SHR.

β_1_- and β_2_AR-selective antagonists alone had no significant effect on the TPR response to tyramine in female rats of either strain. The same was seen in the presence of L-659,066, except for a slight counteracting effect of atenolol (β_1_) in female WKY. These observations indicated that in female rats of both strains one βAR-subtype may substitute for the other and that the β_1_AR had a slightly stronger impact than the β_2_AR. Thus, in both strains, both βAR-subtypes had to be blocked to fully eliminate the βAR-mediated vasodilatory component, which opposed the NE-induced vasoconstriction, regardless of the presence of L-659,066.

### βAR- and α_2_AR-Mediated Control of Vascular Tension in Female Rats Compared to That Previously Observed in Male Rats

In male rats, βAR opposed the TPR response throughout the tyramine-infusion period in WKY but downregulated the TPR response only during the late part of the infusion period in SHR (15). Thus, βAR-mediated vasodilatation played an important role in modulating the TPR response throughout the tyramine-infusion period in both genders in WKY and also in female SHR but played a delayed role in downregulating NE-induced vasoconstriction and TPR in male SHR. This βAR-mediated vasodilatory component may provide protection against development of hypertension in the female SHR, as observed in premenopause women (10).

Similar to that in female WKY (present study), the α_2_AR-antagonist L-659,066 eliminated the vasoconstrictory TPR response to tyramine also in male WKY (3, 4). Nadolol reversed only in part the reduction observed in male WKY (6), different from the full counteraction seen in the female WKY (present study). Thus, the βAR-mediated vasodilation unshielded by α_2_AR inhibition was more efficient in the female than in the male WKY. However, different from the reduced TPR response after pretreatment with L-659,066 seen in the female SHR, L-659,066 had little effect on the TPR response to tyramine in the male SHR, and nadolol did not alter the TPR response in L-659,066-treated male SHR (6). It therefore seemed that α_2_AR-mediated vasoconstriction depended exclusively on βAR-mediated vasodilation as a substrate in the female SHR, whereas the α_2_AR/βAR interaction was totally absent in male SHR.

The ability of female rats of both genders to substitute the effect of one βAR-subtype with that of the other and thus counteract the TPR response to NE was not observed in male rats. In male rats, β_1_-, β_2_-, and β_1+2_AR-antagonists all increased the immediate and late TPR response in WKY and the late response in SHR, with little difference between the different antagonists (15). Similarly, there was no difference in the impact of the β_1_-, β_2_-, and β_1+2_AR-antagonists in male WKY in the presence of L-659,066 (6). Thus, in the males, both β_1and2_AR may contribute to the vasodilatation, but one subtype did not substitute for the other and the two subtypes did not have an additive effect.

### The Impact on the MBP Response to Tyramine in Female Rats Compared to That Previously Observed in Male Rats

The importance of the βAR-mediated vasodilatory component in counteracting the BP response to NE in the female rats was clearly demonstrated by the augmented MBP response to tyramine after nadolol, and the effect of nadolol was far greater in WKY than in SHR. Like for TPR, β_1_- and β_2_AR-selective antagonist had no significant effect on the MBP response, explained by the ability of the two βAR-subtypes to substitute for one another. L-659,066 lowered the MBP response to tyramine in both strains in female rats. In the male rats (4), L-659,066 reduced the response in WKY whereas the reduction was not statistically significant in SHR.

### The β_1_AR–AT1R Interaction in the Control of TPR in Female Rats Compared to That Previously Observed in Male Rats

Losartan alone (3), like atenolol alone, did not alter the TPR response to tyramine-induced NE release in female rats of either strain. However, the combination of the two greatly enhanced the TPR response in the female WKY, similar to that previously observed in male rats of both genders (5). This increased TPR response was likely to result from an increased α_1+2_AR and β_1_AR control of vascular tension in the absence of angiotensin II-AT1R-mediated vasoconstriction. However, in the female SHR, losartan + atenolol reduced the TPR-peak response to tyramine. The mechanism underlying this observation was not clear.

### The Role of βAR and α_2_AR in the Control of HR

As in male rats (15), pretreatment with β_1_AR-antagonists almost totally eliminated the tyramine-induced tachycardia in female WKY and SHR, whereas β_2_AR-antagonist had no effect. This pattern was not different after additional pretreatment with L-659,066 or losartan. These observations showed, as expected, that the β_1_AR dominated the control of HR.

### The Role of βAR and α_2_AR in Tyramine-Induced Salivation

Salivation does not occur in the anesthetized rat unless stimulated, here by the tyramine-induced release of NE. The salivation was mediated through β_1_AR since it was eliminated in all groups given β_1_AR-antagonist as part of the pretreatment in both strains. The same was observed in male rats (T. Berg, unpublished data). Since β_1_AR-antagonists are first-line medications in the treatment of hypertension and cardiac disease, their inhibitory effect on salivation may have deleterious effects on salivary secretion and, thus, oral health. Indeed, xerostomia, hypo-salivation, increased microbiota, and the number of lost teeth were higher in patients on antihypertensive medication than in controls (21). This problem should be given attention particularly in patients on β_1_-blockers in the form of ascertaining adequate oral hygiene.

### Summary and Implications

The present method using tyramine to stimulate the release of NE, allowed a simultaneous study of the effect of βAR and α_2_AR on presynaptic control of catecholamine release and vascular tension. The amount of catecholamines released was most likely much higher than that needed for a full cardiovascular response. Antagonist-induced differences in the TPR response therefore most likely reflected changes due to inhibition of the postsynaptic receptors rather than drug-induced differences in catecholamine release.

The results showed that βAR facilitated tyramine-stimulated NE release in both strains in female rats, similar to previously observed in male rats, and with a greater effect of the β_1_- than the β_2_-subtype in all rats but the male SHR, where the effect of the two subtypes did not differ. α_2_AR-mediated inhibition of NE release did not interfere with βAR-mediated facilitation of release in female rats of both strains or in male WKY, whereas β_1_AR strongly opposed α_2_AR-auto-inhibition of NE release in male SHR. Furthermore, βAR, again with a greater effect of the β_1_- than the β_2_-subtype, opposed α_2_AR-mediated auto-inhibition of the secretion of epinephrine in all rats except the female WKY. The role of β_1_AR in catecholamine release is therefore likely to be important for the antihypertensive effect of β-blockers in both genders. This effect may also have an important therapeutic effect in myocardial ischemia where hypoxia may cause massive release of NE release through NET (12), similar to that induced by tyramine.

It was further concluded that in both genders in WKY and in the female but not in the male SHR, vasoconstrictory α_2_AR and vasodilatory β_1+2_AR reciprocally modulated the α_1_AR-mediated vasoconstriction activated by the tyramine-stimulated release of NE. These results paralleled the observed differences in starting BP in these age-matched rats, with normal BP in both genders in WKY (SBP/DBP = 85/60 and 103/73 mm Hg in female and male WKY, respectively), a moderate hypertension in the female SHR (138/103 mm Hg), and a strongly elevated BP in the male SHR (183/146 mm Hg) (present results for female rats, and (3) for male rats). These results suggested that the females carried a βAR-mediated vasodilatory protection against NE-induced vasoconstriction, which may play a role in maintaining a lower BP in the female gender, even when prone to hypertension as the female SHR. This conclusion is in accordance with the fact that the positive correlation between MSNA and TPR observed in young men was detected in young females only in the presence of the non-selective βAR antagonist propranolol (10). Furthermore, forearm vasoconstriction in response to infused NE was greater in young men than in young women, but after βAR-blockade with propranolol, the vasoconstriction was greater in the women (22). Also the sensitivity to β_2_AR agonist was found to be greater in women than in men. The mechanisms underlying enhanced βAR-dependant vasodilatation in the female are not known. Hormones such as estrogen or progesterone may play a role (23, 24), but other agents may be involved, such as the angiotensin AT2 receptor (25). However, it should also be pointed out that βAR-mediated vasodilatation is opposed by α_2_AR-mediated vasoconstriction. This interaction is evidently sensitive to both strain and gender, from being fully dysfunctional in the male SHR to giving an α_2_AR-induced vasoconstriction even in the presence of β_1+2_AR-blockade in the female WKY, with more or less an equal balance between the impact of the α_2_AR and βAR in female SHR and male WKY. Estrogen has been shown to mobilize one of the three α_2_AR-subtypes, i.e., α_2C_AR, to the surface in VSMC from human, cutaneous arterioles (26), and this may influence the response to NE. The rise in TPR in response to a α_2C_AR-selective agonist was highly variable in female rats but not male rats (3, 11), possibly due to differences in the estrous cycle. It is therefore possible that a change in α_2_AR functionality is the primary change in the gender-dependent difference in the α_2_AR/βAR control of vascular tension.

In the case that βAR-mediated control of TPR and BP is the same in young women as in the young female SHR; some deductions may be made, which may have therapeutic implications. Since L-659,066 reduced the TPR response to tyramine-induced NE release by enhancing βAR-mediated control of TPR in the female but not male SHR, a peripherally restricted α_2_AR-antagonist like L-659,066 may represent a favorable, antihypertensive medication for women. It is important that the α_2_AR-antagonist should not cross the blood–brain barrier, since α_2_AR-agonists such as clonidine, through its central action and inhibition of central sympathetic output, are highly effective antihypertensive medication. A centrally active antagonist may interfere with this antihypertensive mechanism. Since one βAR-subtype may substitute for the other in the female SHR, L-659,066 may be given as an additive to β_1_AR-blocker. A non-selective βAR-antagonist will block any residual βAR-vasodilatation and should therefore be avoided. However, some caution may be in place regarding this combination since L-659,066 combined with atenolol greatly increased the level of circulating epinephrine. The consequences of that, for instance on cardiac function, is not known. In female SHR, losartan + atenolol had little effect on the TPR response to tyramine (present results) but strongly enhanced the vasoconstriction in male rats of both genders (5). It may therefore be suggested that such combination therapy may enhance adrenergic vasoconstriction to a greater extent in men than in women.

## Ethics Statement

All experiments were approved by The Norwegian Animal Research Authority (NARA) and conducted in accordance with the Directive 2010/63/EU of the European Parliament.

## Author Contributions

TB has performed all experiments and data analyses and wrote the manuscript.

## Conflict of Interest Statement

The author declares that the research was conducted in the absence of any commercial or financial relationships that could be construed as a potential conflict of interest.
